# Long-term survival after Carpentier-Edwards Perimount aortic valve replacement in Western Denmark: a multi-centre observational study

**DOI:** 10.1186/s13019-021-01506-x

**Published:** 2021-05-14

**Authors:** Lytfi Krasniqi, Mads P. Kronby, Lars P. S. Riber

**Affiliations:** 1 Department of Cardiac, Thoracic and Vascular Surgery, Odense University Hospital, J. B. Winsløws Vej 4, 5000 Odense C, Denmark; 2 Faculty of Health Science, University of Southern Denmark, Odense, Denmark

**Keywords:** Aortic valve replacement, Carpentier-Edwards Perimount, Long-term survival

## Abstract

**Background:**

This study describes the long-term survival, risk of reoperation and clinical outcomes of patients undergoing solitary surgical aortic valve replacement (SAVR) with a Carpentier-Edwards Perimount (CE-P) bioprosthetic in Western Denmark. The renewed interest in SAVR is based on the questioning regarding the long-term survival since new aortic replacement technique such as transcatheter aortic-valve replacement (TAVR) probably have shorter durability, why assessment of long-term survival could be a key issue for patients.

**Methods:**

From November 1999 to November 2013 a cohort of a total of 1604 patients with a median age of 73 years (IQR: 69–78) undergoing solitary SAVR with CE-P in Western Denmark was obtained November 2018 from the Western Danish Heart Registry (WDHR). The primary endpoint was long-term survival from all-cause mortality. Secondary endpoints were survival free from major adverse cardiovascular and cerebral events (MACCE), risk of reoperation, cause of late death, patient-prothesis mismatch, risk of AMI, stroke, pacemaker or ICD implantation and postoperative atrial fibrillation (POAF). Time-to-event analysis was performed with Kaplan-Meier curve, cumulative incidence function was performed with Nelson-Aalen cumulative hazard estimates. Cox regression was applied to detect risk factors for death and reoperation.

**Results:**

In-hospital mortality was 2.7% and 30-day mortality at 3.4%. The 5-, 10- and 15-year survival from all-cause mortality was 77, 52 and 24%, respectively. Survival without MACCE was 80% after 10 years. Significant risk factors of mortality were small valves, smoking and EuroSCORE II ≥4%. The risk of reoperation was < 5% after 7.5 years and significant risk factors were valve prosthesis-patient mismatch and EuroSCORE II ≥4%.

**Conclusions:**

Patients undergoing aortic valve replacement with a Carpentier-Edwards Perimount valve shows a very satisfying long-term survival. Future research should aim to investigate biological valves long-term durability for comparison of different SAVR to different TAVR in long perspective.

## Background

Aortic stenosis (AS) is the most frequent primary valve disease in Europe and North America [1]. Surgical intervention remains the gold standard in treating this condition [2]. Surgical aortic valve replacement (SAVR) with bioprosthetic is in Europe usually offered to patients older than 65 years of age with severe AS or severe aortic valve regurgitation and suitable for surgery, though it also is considered in patients younger than 65 years of age in preference of mechanical valves, depending on life expectancy, comorbidity, compliance and patient preference [1].

The greatest weakness of bioprosthetic valves is their limited durability, as they are all in time subject to structural valve deterioration (SVD; calcification, pannus formation, cusp tears, or flail) as well as the risk of non-SVD (regurgitation, prosthesis malposition, patient-prosthesis mismatch, late embolization, and prosthetic endocarditis) which applies to all valves [1, 3–7]. The rate of SVD correlates with age, degenerating faster in younger patients [1, 6, 8–10]. Patients operated with SAVR have several risks of postoperative complications [11] including, but not limited to, acute renal failure [12, 13], major life-threatening bleeding [12, 14, 15], permanent pacemaker [12–15], postoperative atrial fibrillation (POAF) [12, 16–18] and stroke [12–14]. The operative mortality of SAVR is estimated between 2.8 and 4% [1, 9, 14, 19]. The Carpentier-Edwards Perimount (CE-P) (Edwards Lifesciences, Irvine, CA, USA) valve was introduced in Denmark 1999 and is now the preferred valve in multiple centres due to very satisfying manufacture-related durability [19].

The future application of SAVR is however being questioned, due to the technological advancement of transcatheter aortic-valve replacement (TAVR) [20]. The primary objective of this registry-based retrospective cohort study is to investigate the long-term survival of all patients who underwent a solitary SAVR with CE-P valve bioprosthesis and secondary to explore the adverse clinical outcomes.

## Methods

This registry-based study was approved by the Region Southern Denmark Data Protection Agency, Odense, Denmark, and the study followed the Strengthening the Reporting of Observational Studies in Epidemiology (STROBE) reporting guideline.

We conducted a population-based retrospective cohort study analysis including all patients who underwent solitary SAVR with CE-P valve in Western Denmark based on data from November 1999 to November 2013. The primary endpoint of this study was long-term survival from all-cause mortality with valve size 19 to 29 mm in comparison with the Danish background population. Secondary endpoints were survival from major adverse cardiovascular and cerebral events (MACCE), risk of reoperation, cause of late death, valve patient-prothesis mismatch (VP-PM), PAOF, risk of AMI, stroke and pacemaker. We excluded patients operated with other valve types of aortic valve replacement (AVR) or had concomitant surgery.

**Fig. 1:**
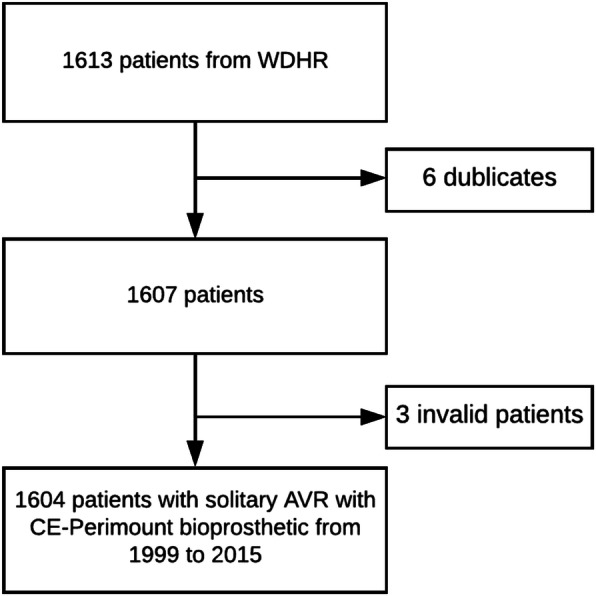
Flow chart of study population selection. WDHR, Western Danish Heart Registry; AVR, aortic valve replacement; CE-Perimount, Carpentier-Edwards Perimount

The data was obtained November 2018 from the Western Danish Heart Registry (WDHR), The National Danish Patient Registry and the Program of clinical quality development of the Danish Regions. WDHR is a multi-centre prospective registry and is the most comprehensive registry regarding AVR in Denmark. The background population was obtained from Statistics Denmark and matched according to our cohort with a mean age of 73 years and a follow-up of 15 years. MACCE is defined as all-time cardiovascular events and early (≤30 days) cerebrovascular disease. Low-risk patients was defined according to guidelines, EuroSCORE II < 4% [1]. VP-PM was evaluated as effective orifice area index (EOAI) < 0.85 cm^2^/m^2^ estimated with manufactural effective orifice area (EOA) measurements indexed with perioperative body surface area (BSA) knowing that the measurements reliability to predict EOAI are questioned. A total of 1613 people were registered with solitary SAVR in WDHR (Fig. 1). Six patients were duplicates, so the first surgery where kept, and the late will be included as reoperation. Three patients were removed due to invalid or inactive social security number. That leaves a population of 1604 patients whom all had an efficient follow-up.

Baseline characteristics were described using means, range, standard deviation, for continuous variables, median and quartiles for skewed variables and proportions for categorical variables. Time-to-death was calculated as the time in years from the date of operation to the time of death from all-cause or MACCE-cause. Survival was performed with the use of Kaplan–Meier estimates. The overall survival curve was compared to the background population graphicly by evaluating overlap of curves and confidence interval. Log-rank test was performed to compare equality of survivor function of subgroups. Cumulative incidence function of reoperation was performed with Nelson-Aalen cumulative hazard estimates. Only the first re-event for each patient during the study was used for analysis. Cox regression was used to detect the risk factors for death and reoperation of any cause reporting hazard ratio (HR) and 95% confidence interval (CI). The age between 65 to 70 was set as reference for age related cox analysis. The same applies to prosthesis size 25 in respect to valve size related cox analysis. A *P*-value of < 0.05 was considered significant. All statistical analyses were performed with the use of STATA software, version 15.

## Results

We included 1604 patients who received a solitary SAVR with CE-P bioprosthesis between November 1999 and November 2013, with a total follow-up of 11.386 patient-years (median follow-up: 7.1 years). For the same period, 2880 non-solitary SAVR were performed, 987 solitary AVR with different types of bioprosthetic valves than CE-P (i.e., Mitroflow) and 511 solitary AVR with mechanical valves.

### Baseline and operative characteristics

Table 1 summarize the baseline and operative characteristics. The median age was 73 years (IQR: 69–78); with 19, 167, 285, 846 and 287 patients with the age of < 50, 50–65, 65–70, 70–80 and ≥ 80 years, respectively. The cohort was 61.3% male; AS was the most common aetiology for SAVR (75.1%); mean ejection fraction was 53%; mean EuroSCORE II (ES2) was 1.45% with a range of 0.5–39.62%; 6.4% received a size 19 valve; 21.6% received a size 21 valve; 35.2% received a size 23 valve; 36.7% received a size ≥25 valve; 87.3% had an EOAI ≥0,85; the mean BSA was 1.89 m^2^ (range 1.34–2.67 m^2^); and 12.7% (*n* = 188) was evaluated with VP-PM. The mean hospital stay was 8 days (median: 6 days, *n* = 1520) for all and 10 days for ES2 ≥ 4% (median: 8 days).

**Table 1:** Preoperative baseline characteristics of patients undergoing solitary SAVR with CE-Perimount bioprosthesis

Patients, n	1604
Male, n (%)	984 (61.3%)
Age	
Median (IQR)	73 (69–78)
Mean, years (Range)	72.7 (20–91)
Etiology, n (%)	
Aortic Stenosis	1204 (75.1%)
Aortic Insufficience	248 (15.5%)
Stenosis and Insufficience	7 (0.4%)
Endocarditis	30 (1.9%)
Others incl. Missing	115 (7.2%)
Valve size, *n* = 1601 (%)	
19 mm	103 (6.4%)
21 mm	346 (21.6%)
23 mm	564 (35.2%)
25 mm	412 (25.7%)
27 mm	156 (9.7%)
29 mm	20 (1.3%)
EOAI ≥0,85 (%), *n* = 1483	1295 (87.3%)
Ejection Fraction, *n* = 1469	
Mean % (95% CI)	53 (52.55–53.89)
Range	0–92
Smoking, *n* = 1473	
Active smoker	237 (14.8%)
Non-smoker	524 (32.8%)
Previous smoker	712 (44.6%)
EuroSCORE II, *n* = 1604	
Mean ± SD (95% CI)	1.45 ± 2.08 (1.35–1.55)
Range, score	0.5–39.62
Diabetes mellitus, *n* = 1561 (%)	204 (13.1%)
Previous AMI, *n* = 1557 (%)	119 (7.6%)
Previous PCI, *n* = 1555 (%)	110 (7.1%)
Lipid-lowering treatment, *n* = 1538 (%)	707 (46%)
Hypertension treatment, *n* = 1528 (%)	873 (57.1%)
Previous Operations, *n* = 1602 (%)	
0	1476 (92.1%)
Previois aortic valve operation	11 (0.7%)
Other heart surgery	39 (2.4%)
BSA, *n* = 1486	
Mean ± SD, m^2^ (95% CI)	1.89 ± 0.2 (1.88–1.90)
Range, m^2^	1.34–2.67

*BSA* Body surface area, *EOAI* effective orifice area index, *IQR* Interquartile range;

### Early mortality and morbidity

In-hospital mortality was 2.7% (*n* = 43) and early (≤30 days) mortality at 3.4% (*n* = 55) in the entire cohort, 3.1% for low-risk patients (ES2 < 4%) and 11.9% for ES2 ≥ 4%. The incidence of POAF was observed at 36.4% (*n* = 583), stroke at 1.3% (*n* = 21) and for AMI 0.4% (*n* = 7) with hazard ratio (HR) at 1.03 (*P* = 0.70, 95% CI: 0.89–1.20), 1.35 (*P* = 0.32, 95% CI: 0.74–2.45) and 1.67 (*P* = 0.31, 95% CI: 0.63–4.47), respectively. The incidence of postoperative treatment (< 30 days) with permanent pacemaker or ICD was at 3.9% (*n* = 62).

### Survival

**Fig. 2:**
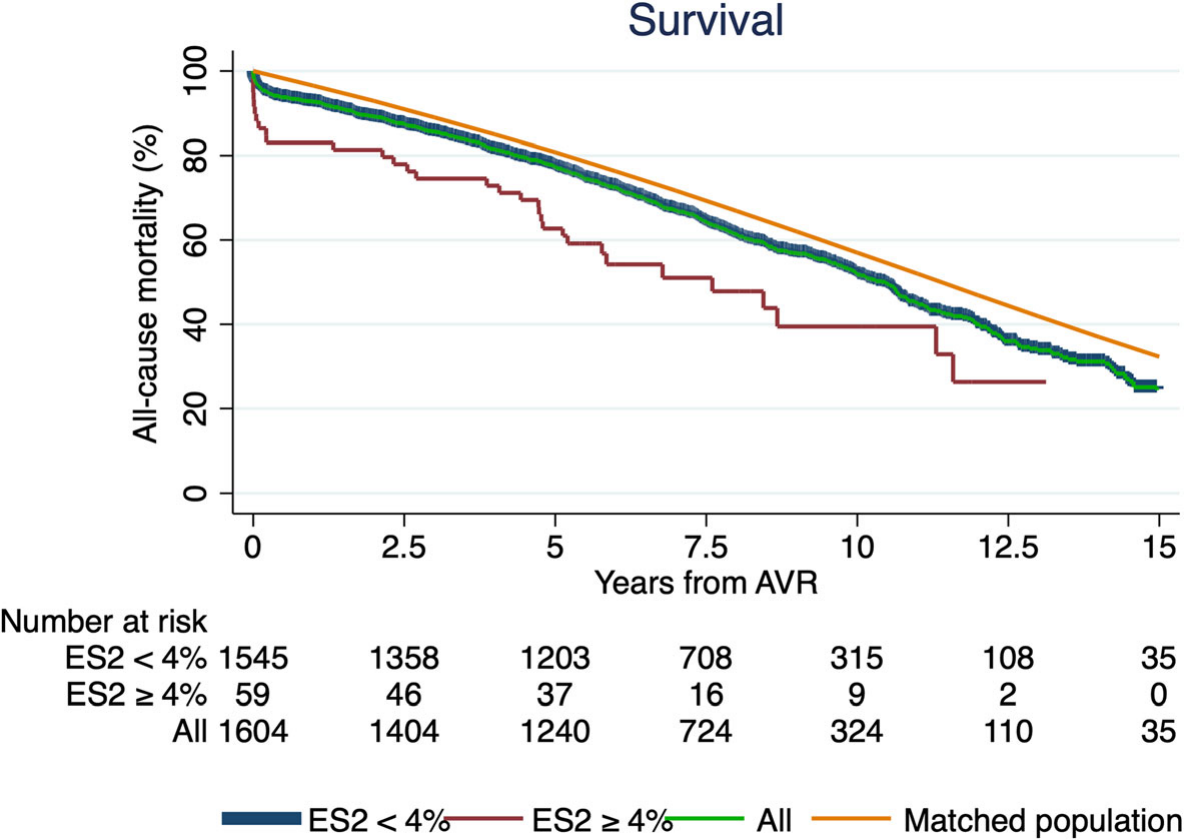
Survival from all-cause mortality of all patients after aortic valve replacement (green line) compared with normal Danish population (orange line) and according to EuroSCOREII (ES2). Green and blue line are very overlapped. Survival for all patients at 1-, 5-, 10- and 15-year from was 93, 77, 52 and 24%, respectively. The 5-, 10-, and 15-year survival for low-risk patients was 78, 52 and 25%, respectively, and for ES2 ≥ 4, 63 and 39% at 5 and 10 years, respectively. The survival of the matched background population in Denmark was 81, 57 and 32% after 5-, 10- and 15-years, respectively

**Fig. 3:**
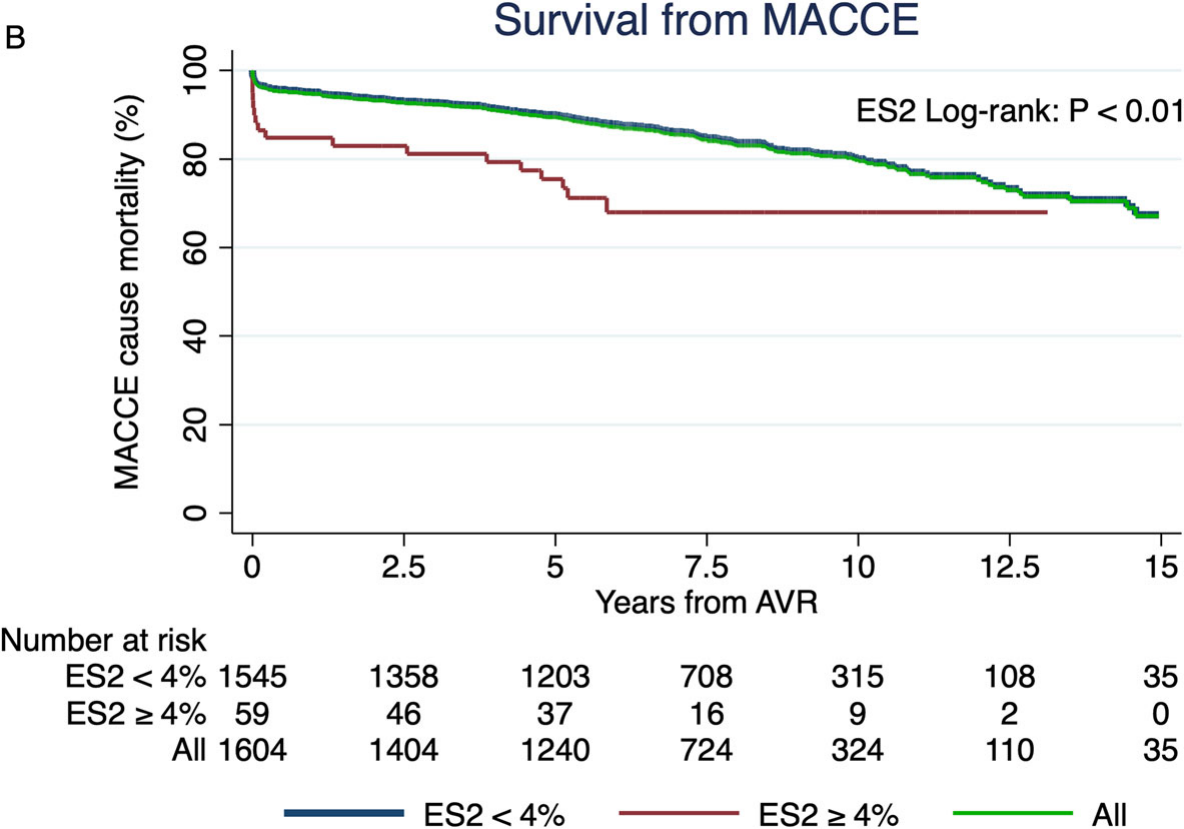
Survival from major adverse cardiovascular and cerebral events (MACCE) and according to EuroSCORE II (ES2). Log-rank test of ES2 < 4% (blue) and ES2 ≥ 4% (red). The overall 5-, 10- and 15-year survival from MACCE-caused mortality (green) was 89, 80 and 67%, respectively

**Fig. 4:**
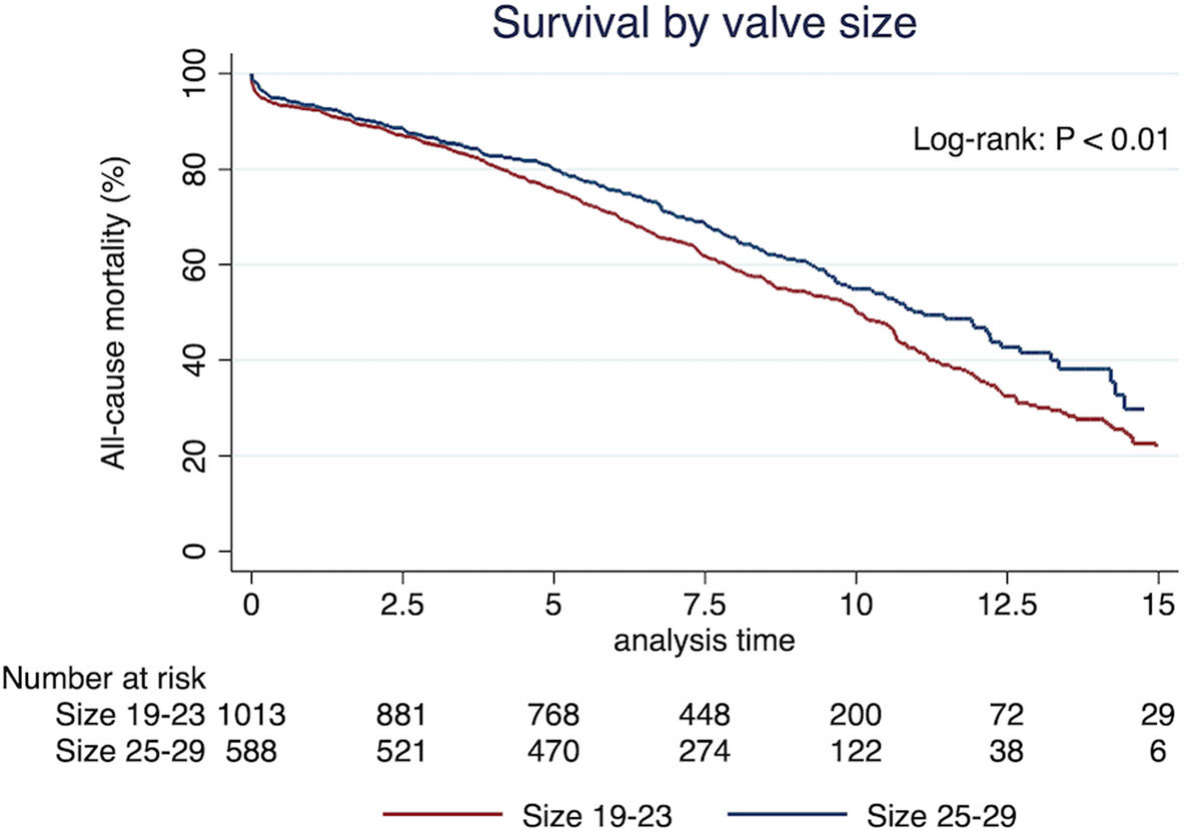
Survival according to prosthesis sizes 19–23 (red) and 25–29 (blue). The 1-, 3-, 5-, 10- and 15-year survival for valve 19–23 was 92.4, 85.2, 75.8 and 21.9%, respectively and for size 25–29, 93.2, 86.5, 79.9, 54.9 and 29.7%, respectively. The number of patients presented with VP-PM was 155 and 33 for valve 19–23 and 25–29, respectively

Figure 2 shows Kaplan-Meier survival plot for the CE-P bioprosthesis. The 1-, 5-, 10- and 15-year survival from all-cause mortality was 93, 77, 52 and 24%, respectively. The number of patients follow-up after 1, 5, 10 and 15 years were 1487, 1240, 324 and 35, respectively. The 5-, 10-, and 15-year survival for low-risk patients (*n* = 1545) was 78, 52 and 25%, respectively, and for ES2 ≥ 4, *n* = 59) 63 and 39% at 5 and 10 years, respectively. The survival of the matched background population in Denmark was 81, 57 and 32% after 5-, 10- and 15-years, respectively. The survival curve and the matched population were significantly different over the 15 years of follow-up (*P* < 0.05). There was great overlap of the two populations with no difference in 10-year survival when censoring all patients with early death (*n* = 55). Survival in relations to age after 10 years was 75, 68, 67, 50 and 32% for ages < 50, 50–65, 65–70, 70–80 and ≥ 80 years, respectively. The 15-year survival of patients between 50 and 65 and 65–70 was 53 and 49%, respectively. The 5-, 10- and 15-year survival from MACCE-caused mortality was 89, 80 and 67%, respectively (Fig. 3). The subgroups survival by ES2 are illustrated in Fig. 3 (log-rank: *P* < 0.05). Figure 4 presents survival according to prosthesis size. Size 25–29 are grouped due to overlap of graphs when estimated separately. Patients implanted with a size 19 had the worst outcome, but since the sample size was only 103, they were grouped to 19–23. The 1-, 3-, 5-, 10- and 15-year survival for valve 19–23 was 92.4, 85.2, 75.8 and 21.9%, respectively and for size 25–29, 93.2, 86.5, 79.9, 54.9 and 29.7%, respectively. The median survival in patients with prosthesis sizes 25–29 was 7.2 years and for size 19–23 it was 7 years, while size 19 was 6.5 years. The number of patients presented with VP-PM was 155 and 33 for valve 19–23 and 25–29, respectively. Using Cox regression analysis of long-term survival (Table 2), smoking, age 70–80, age ≥ 80, and valve size 19–23 were significant risk factors with a HR of 1.59 (*P* < 0.01), 1.81 (*P* < 0.01), 3.19 (*P* < 0.01) and 1.22 (*P* = 0.03), respectively. ES2 < 4% had a HR of 0.61 (*P* < 0.01) while ES2 ≥ 4% had a HR of 1.64 (*P* < 0.01). Male gender, diabetes mellitus, prior smoker and valve prosthesis patient mismatch had a HR between 1.07 and 1.22 but were non-significant. Post hoc analysis of lipid-lowering treatment correlated with decreased risk of death the first 10 years (HR: 0.68; *P* < 0.01). The cause of death in the cohort was due to MACCE (36.2%, *n* = 273); Cancer or tumor (19.4%, *n* = 146); Pulmonary failure (8.1%, *n* = 61); Late cerebrovascular disease (7.7%, *n* = 58); External cause of injury (3.4%, *n* = 26); Miscellaneous (17%, *n* = 128); Unknown (8.2%, *n* = 62).

**Table 2:** Cox regression analysis of death and reoperation

	Death		Reoperation	
	Hazard Ratio (95% CI)	*P*-value	Hazard Ratio (95% CI)	*P*-value
Male gender	1.07 (0.92–1.24)	0.360	1.56 (0.91–2.66)	0.107
Age group^a^				
65–70	1.0		1.0	
< 50	1.35 (0.55–3.33)	0.516	5.61 (1.59–19.8)	0.007*
50–65	1.05 (0.74–1.51)	0.77	2.44 (1.17–5.08)	0.017*
70–80	1.81 (1.43–2.28)	< 0.001*	0.71 (0.37–1.38)	0.312
≥ 80	3.19 (2.46–4.12)	< 0.001*	0.60 (0.21–1.70)	0.337
Reoperation^b^	1.06 (0.74–1.52)	0.747		
Valve size				
25	1.0		1.0	
19–23	1.22 (1.03–1.45)	0.025*	0.73 (0.42–1.26)	0.252
27–29	0.94 (0.71–1.25)	0.658	1.03 (0.45–2.34)	0.948
Diabetes Mellitus	1.22 (0.99–1.51)	0.067*	1.38 (0.68–2.81)	0.369
Smoker	1.59 (1.29–1.97)	< 0.001*	1.61 (0.74–3.52)	0.234
Previous smoker	1.08 (0.91–1.28)	0.383	1.39 (0.77–2.51)	0.269
VP-PM	1.18 (0.95–1.48)	0.144	2.19 (1.21–3.98)	0.010*
EuroSCORE II < 4%	0.61 (0.43–0.87)	0.006*	0.12 (0.06–0.22)	< 0.001*
Hypertension treatment	1.01 (0.87–1.17)	0.889	0.98 (0.59–1.64)	0.945
Lipid-lowering treatment^c^	0.68 (0.58–0.80)	< 0.001*	1.09 (0.61–1.96)	0.759

*VP-PM* Valve prosthesis-patient mismatch. *P < 0.05

^a^Age groups for reoperation are sorted by the age of the primary CE-P procedure

^b^Reoperation after primary AVR with another CE-P or TAVR

^c^Cox regression of the first 10 years

### Reoperation

**Fig. 5:**
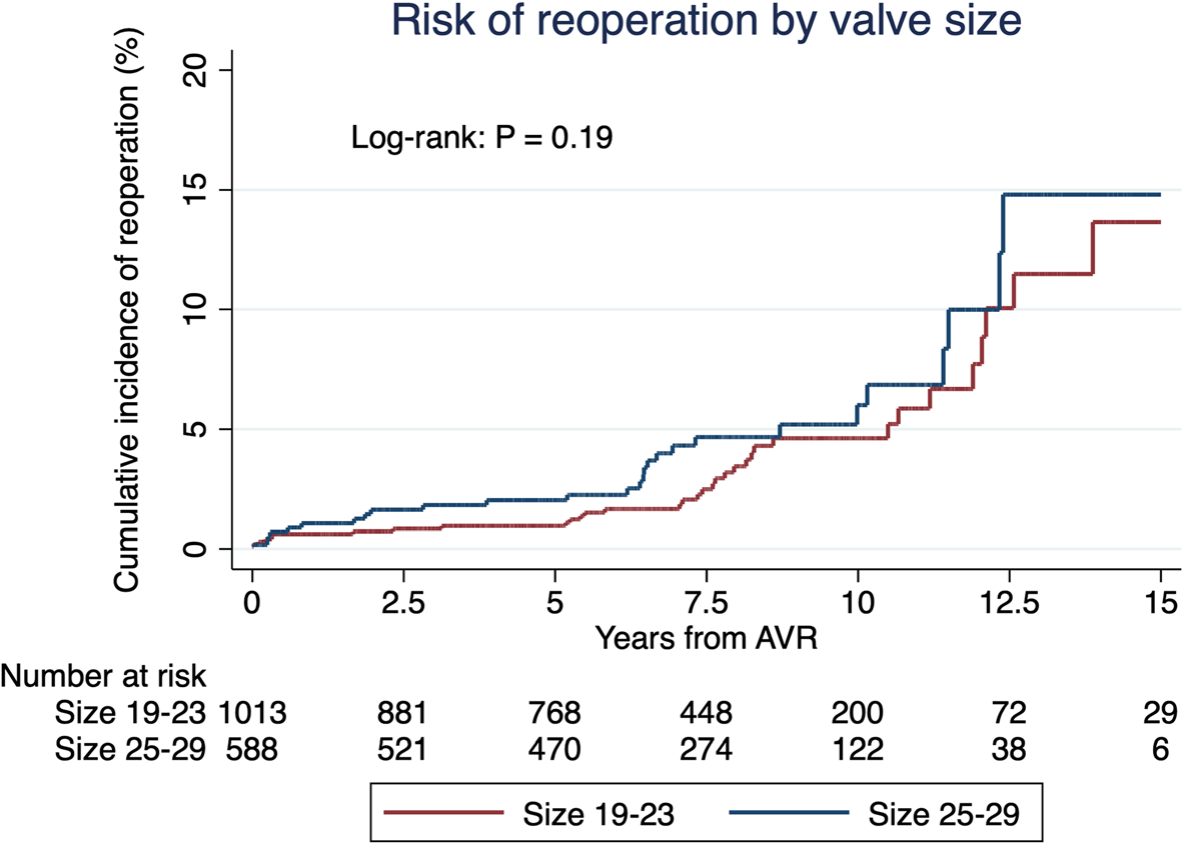
Cumulative incidence of reoperation according to prosthesis sizes 19–23 (red) and 25–29 (blue). Log-rank test of sizes 19–23 (red) and 25–29 (blue). The risk of reoperation at 7.5 years for the sizes 19–23 and 25–29 is 2.46 and 4.57%, respectively

The risk of reoperation with a new CE-P or TAVR of any cause during the follow-up was 4% (*n* = 64) and 22% (*n* = 14) was due to endocarditis. The cumulative incidence of reoperation according to prosthesis size are presented in Fig. 5 and Table 3. The risk of reoperation for all prosthesis sizes after 5, 10 and 15 years was 1.3, 5 and 13.3%, respectively. The risk of reoperation at 7.5 years for the sizes 19–23 and 25–29 is 2.46 and 4.57%, respectively (log-rank *P* = 0.19). Cox regression analysis of reoperation indicated that age < 50, age 50–65, and VP-PM was a significant risk factor (HR: 5.61, *P* < 0.01, HR: 2.44, *P* < 0.02, HR: 2.19, *P* = 0.01, respectively), and ES2 < 4% was significant correlated with lower risk (HR: 0.12, *P* < 0.01). ES2 ≥ 4% was significantly correlated with a higher risk of reoperation (HR: 8.6, *P* < 0,01).

**Table 3:** Cumulative incidence function of reoperation

Prosthesis size	CIF (95% CI) at year 1	CIF (95% CI) at year 5	CIF (95% CI) atyear 7,5	CIF (95% CI) atyear 10	CIF (95% CI) at year 15
19–23	0.62% (0.3–1.4)	0.95% (0.5–1.8)	2.46% (1.5–4.0)	4.52% (3.0–6.8)	12.8% (7.9–20.6)
25–29	1,06% (0.5–2.4)	2.01% (1.1–3.6)	4.57% (2.9–7.1)	5.84% (3.7–9.2)	13.9% (8.0–23.5)
All	0.78% (0.4–1.4)	1.34% (0.9.-2.1)	3.24% (2.3–4.5)	5.0% (3.7–6.8)	13.3% (9.2–19.2)

*CI* Confidence interval, *CIF* Cumulative incidence function

## Discussion

This multi-centre observational follow-up study evaluated up to 15 years of long-term survival after SAVR with CE-P valve in terms of mortality and clinical outcomes in 1604 Danish patients having a solitary SAVR. Our study found a 30-day all-cause mortality at 3.4%, and a 5-, 10- and 15-year survival from all-cause at 77, 52 and 24%, respectively including a 7.5-year risk of reoperation < 5%.

This study’s observation of all-cause mortality was consistent with the Danish matched population after early risk and until year 10, were the mortality takes another discrete yet noticeable hit until the end of the follow-up (Fig. 2). The early mortality was higher than predicted in comparison to mean ES2 of the population (meanES2: 1.45%, predicted number of deaths: 23). After patients surpass the general risk of the operation the first 30 days, the survival rate matches to the Danish population for 10 years. We may assume that the CE-P valves durability is being challenged by this point, which can also be supported by literature describing the development of SVD in CE-P at 9.24% after 10 years [10], and 18% after 15 years [7]. The Danish population is matched according to the mean age of our baseline (Median: 73 years, range: 20–91) which matches previous reports [3, 7–9, 19, 21, 22], and 12% of the cohort being < 65 years. SAVR at the age of 20–30 is very rare and the choice of valve should consider the risk of lifelong anticoagulation with mechanical valve versus the very high risk of reoperation with bioprosthetic. Young age and small valve sizes are well associated with accelerated development of SVD, why they are usually recommended mechanical valves, especially under the age of 60 [1, 3, 6, 8–10]. Our data suggests that age < 65 years is significantly associated with reoperation, however not with death (Table 2). However, the risk of death was significantly increased for smaller valve size 19–23 why the biggest valve possible including root-enlargement must be considered when the patient is in the range of 19–23 mm to avoid VP-PM. Furthermore, reoperation has a HR of death at 1.07 (*P* = 0.75) and older demographics (≥70 years) have a significant increased risk of death and decreased risk of reoperation (Table 2). This may suggest that ether the durability of the CE-P valve is not the primary cause of death in this age group, or and more likely that they’re not suitable for reoperation because of development and progress of comorbidities. Furthermore, that the increased risk in mortality in small vales is presumable explained by the demographics rather than SVD development. Nevertheless, our findings could indicate that patients undergoing SAVR with CE-P can expect a survival matching the background population the next 10 years after overcoming early risks. A Danish study described the 10-year survival from all-cause mortality of CE-P at 36% resulting, which is significantly worse than our findings of 52% for roughly the same period [19]. This could be explained by their single-centre approach, their inclusion of concomitant CABG procedures and the fact that we conducted our results November 2018 which prolongs our follow-up with 4.5 years. The surgical technique and postprocedural medical attention have improved tremendously over time, and therefore a prolonged period of 4.5 years will have a significant effect on a 10-year measurement. Additionally, we may assume that if patients are eligible for concomitant CABG, they have increased probability of comorbidity contributing to the risk of death. It does however interfere with their true long-term survival analysis and durability of the CE-P valve. It is noteworthy that our survival is consistent with other studies; overall 5-, 10- and 15-year survival rate in China (81.58 66.19 and 57.33% [10]), overall 10- and 15-year survival rate in France (52.4 and 31.1% [9]), overall 5-, 10- and 15-year survival rate in a Canadian study (78, 55 and 34% [21]) and others [23]. We also investigated the survival from MACCE-cause mortality in order to more comprehensive describe the valve durability in combination with all causes of late death (Fig. 3). The cause of late death was 55.6% due to other than valve or MACCE-related causes, why one might argue that CE-P surpasses the general patient’s life expectancy and therefore making it a reliable choice for SAVR and that future randomized studies could take MACCE into account when evaluating the durability in order to give a more widespread evaluation of the valves. VP-PM (HR: 2.19) and high-risk patients (HR: 6.62) had a significant impact on the risk of reoperation, while valve size was not an independent risk factor. VP-PM’s impact on reoperation contradicts earlier report of CE-P using calculated EOA reference values to predict VP-PM, which were substantially higher than the values provided by the manufacture [24]. The prediction of VP-PM is controversial because of various calculations. However, one study comparing 4 methods argued that the best calculations are made by EOA measured in vivo by Doppler echocardiography [25]. The latest meta-analysis supported the association of VP-PM have higher risk for perioperative, 1-, 5- and 10-year mortality rates in comparison to those with non-significant or no VP-PM. In our study, 12.7% had EOAI < 0.85 why this could contribute to MACCE-cause mortality, though our estimates are based on manufactural EOA measurements indexed with BSA. ES2 ≥ 4% were found as an independent risk factor for reoperation, which correlates with the selection-bias in this group. Patients with ES2 ≥ 4% have more comorbidities such as renal failure, previous AMI, COPD, poor lung capacity, endocarditis or the need of acute need of operation. This could also explain the prolonged hospital stay in high risk compared with low risk. The small valve sizes were not associated with reoperation, which may be explained by unsuitability for surgery or the small sample size. The operative risk of reoperation due to SVD in CE-P was earlier described at 0% for size 19 and 21 and 0.1% for all valve sizes [19], which may support our non-correlation of small valves with reoperation. However, the only way to finally evaluate whether a correlation between small valves and the development of SVD is present, is by performing a prospective study with close echocardiographic follow-up, so no patient would experience SVD without notice. The cumulative incidence function of reoperation due to any cause was < 5% after 7.5 years (Table 3), which is more than previous reports of freedom of reoperation at 96–99.5% after 10 years [19, 21, 26]. This could be due to our broad range of age (20–91 years) [21]. Forcillo et al. divided their population into 3 different age-categories illustrating that younger patients had a higher risk of reoperation with a 10-year risk of 90% in < 60 years of age with no mention of the subgroup size [21], and Langanay et al. had an age range of 80–96 years [26]. The small sample size after 7.5 years inhibits further associations to be made. Other clinical implications such as stroke, AMI, permanent pacemaker or ICD and POAF is at focus, especially in the comparison to TAVR. We observed a 30-days stroke rate at 1.3% (21 patients; HR:1.35; CI:0.74–2.45; *P* = 0.32) being lower than the SAVR findings in the PARTNER 3 trail which was 2.4% (meanES2: 1.5%) [20]. The 30-day incidence of AMI was also lower in our study (0.4%) compared to literature (1.3–1.6%) [20, 27]. Lower incidence was also found regarding pacemaker or ICD implantation (3.9%), as SAVR-reports once again reports higher incidences (4.1–7.4% - pacemaker only) [20, 27, 28]. Interestingly, our data matches in general the findings found in another CE-P valves cohort study (stroke 1%, AMI 1%, atrioventricular-block 4% [21]). The incidence of POAF (36.4%) is however higher than some reports (11%) [27], which might be due to the definition of POAF in regards to duration and the overall awareness in the ward, since it is comparable to other findings (32–39.5%) [20, 29].

With survival matching the background population after overcoming the early risks, along with the lower risk of AMI, stroke, pacemaker/ICD compared to the literature, and only 36.2% of the mortality is caused due to major adverse cardiovascular and cerebral events, our data strongly suggest that the CE-P valve have favourable characteristics with moderated cardiovascular risks compared to other bioprosthetic valves i.e. Mitroflow valve [3].

This research was limited by its retrospective design and the absence of continuance echocardiographic evaluation of SVD [6, 30]. Long-term SVD and its relations to long-term survival therefore still remains in question. The long-term data is also limited given that only 20% of the cohort had a flow-up for more than 10 years available. The VP-PM data is also limited by calculations based on manufactural EOA, and can therefore only be acknowledged as suggestive. Furthermore, of the 1604 patients, 754 died before this study was conducted, and postoperative evaluation of SVD would not have been possible and 8.2% of cause of death remains uncertain. Additionally, the cohort was database-dependent and therefore relays on correct SKS codes, though earlier studies of the WDHD have shown very good quality evaluation with errors lower than 3% [31]. Selection bias in this study was minimized by the universal health care system based on the Beveridge model.

## Conclusion

We cautiously conclude that our findings match previous literature, and that CE-P is a well-tested valve with satisfying long-term durability, which do not affect the long-term survival seriously for patients undergoing aortic valve replacement. This support the idea for future comparative research of TAVR-valves may be randomized solely against Carpentier-Edwards Perimount, and not pooled with other inferior bioprosthetic valves [3].

## Data Availability

The datasets used and/or analysed during the current study are available from the corresponding author on reasonable request.

## Abbreviations

AS: Aortic stenosis
AVR: Aortic valve replacement
BSA: Body surface area
CE-P: Carpentier-Edwards Perimount
CI: Confidence interval
EOA: Effective orifice area
EOAI: Effective orifice area index
ES2: EuroSCORE II
HR: Hazard ratio
MACCE: Major adverse cardiovascular and cerebral events
POAF: Postoperative atrial fibrillation
SVD: Structural valve deterioration
SAVR: Surgical aortic valve replacement
TAVR: Transcatheter aortic-valve replacement
VP-PM: Valve patient-prothesis mismatch
WDHR: Western Danish Heart Registry
